# Tissue ACE phenotyping in prostate cancer

**DOI:** 10.18632/oncotarget.27276

**Published:** 2019-10-29

**Authors:** Sergei M. Danilov, Alexey V. Kadrev, Olga V. Kurilova, Victoria E. Tikhomirova, Olga V. Kryukova, Vadim N. Mamedov, David M. Kamalov, Natalia V. Danilova, Dmitry A. Okhobotov, Nurshat M. Gayfullin, Valery V. Evdokimov, Boris J. Alekseev, Olga A. Kost, Larisa M. Samokhodskaya, Armais A. Kamalov

**Affiliations:** ^1^ Department of Medicine, Division of Pulmonary, Critical Care, Sleep and Allergy, University of Illinois at Chicago, IL, Chicago, USA; ^2^ Department of Medicine, University of Arizona, Tucson, AZ, USA; ^3^ Medical Research and Educational Center, Lomonosov Moscow State University, Moscow, Russia; ^4^ Department of Chemistry, Lomonosov Moscow State University, Moscow, Russia; ^5^ Faculty of Fundamental Medicine, Lomonosov Moscow State University, Moscow, Russia; ^6^ Lopatkin Research Institute of Urology and Interventional Radiology, Moscow, Russia

**Keywords:** angiotensin I-converting enzyme, prostate cancer, benign prostate hyperplasia, CD143, monoclonal antibodies

## Abstract

Epithelial cells of prostate express significant level of ACE and, as a result, seminal fluid has 50-fold more ACE than plasma. The substitution of highly specialized prostate epithelial cells by tumor cells results in dramatic decrease in ACE production in prostate tissues. We performed detailed characterization of ACE status in prostate tissues from patients with benign prostate hyperplasia (BPH) and prostate cancer (PC) using new approach- ACE phenotyping, that includes evaluation of: 1) ACE activity with two substrates (HHL and ZPHL); 2) the ratio of the rates of their hydrolysis (ZPHL/HHL ratio); 3) the ratio of immunoreactive ACE protein to ACE activity; 4) the pattern of mAbs binding to different epitopes on ACE – ACE conformational fingerprint - to reveal conformational changes in prostate ACE due to prostate pathology. ACE activity dramatically decreased and the ratio of immunoreactive ACE protein to ACE activity increased in PC tissues. The catalytic parameter, ZPHL/HHL ratio, increased in prostate tissues from all patients with PC, but was did not change for most |BPH patients. Nevertheless, prostate tissues of several patients diagnosed with BPH based on histology, also demonstrated decreased ACE activity and increased immunoreactive ACE protein/ACE activity and ZPHL/HHL ratios, that could be considered as more early indicators of prostate cancer development than routine histology. Thus, ACE phenotyping of prostate biopsies has a potential to be an effective approach for early diagnostics of prostate cancer or at least for differential diagnostics of BPH and PC.

## INTRODUCTION

Prostate cancer (PC) is the second most common cause of cancer related death for men - worldwide more than a million men are diagnosed with PC each year and about 1/3 of them would die from this disease [1]. The prostate-specific antigen (PSA) is the most common tumor marker for the early detection of PC. The PSA analysis used today, including its modifications (free PSA, ratio of free PSA to total), has low specificity and sensitivity and depends on many factors including PSA elevation with benign prostate hyperplasia (BPH), acute prostatitis [2–4], and even with the manipulation of the prostate like prostate massage [5]. As a result of low specificity of PSA test, the biopsy is positive in only about 1/3 of patients with PSA elevation between 4 and 10 ng/ml [6] or between 2 and 10 ng/ml [7] and overdiagnosis and overtreatment of PC affect up to 50% of new diagnoses [8]. Markers like proPSA or PSA3 use other identification principles and have more accurate diagnostic value, but due to higher cost and low availability are performed by a small number of laboratories [9]. Therefore, new prostate cancer screening markers with high specificity and sensitivity are still required [2, 9, 10].

Angiotensin I-converting enzyme (ACE, CD143, EC 3.4.15.1), a Zn^2+^-dependent peptidyldipeptidase with two catalytic centers [11], is a key regulator of blood pressure which also participates in the development of vascular pathology and remodeling [12, 13]. There are several reasons why deep study of ACE in prostate could be beneficial for the putative early detection of prostate cancer:

1) Since 1998 [14], ACE (or, more correctly, ACE inhibitors, ACEI) received considerable attention in oncology as preclinical and clinical data suggested that ACEI may potentiate the effect of certain systemic antitumor therapies [reviewed in details in 15]. The use of ACE inhibitors was associated with better outcomes in patients with different tumors (including prostate cancer) who were receiving chemotherapy or anti-VEGF therapy [reviewed in 15].

2) Glandular epithelial cells of prostate express significant level of ACE and, therefore, seminal fluid has 50-fold more ACE concentration than plasma [16–18]. The substitution of highly specialized prostate epithelial cells by tumor cells results in dramatic decrease in ACE activity in prostate tissues [19, 20], therefore ACE phenotyping of prostate biopsies may have clinical (diagnostic) significance.

3) Our studies with monoclonal antibodies (mAbs) to numerous conformational epitopes on the surface of human ACE revealed that the pattern of mAb binding to ACE - “conformational fingerprint of ACE” – is tissue specific, mainly due to tissue specific ACE glycosylation [21–25]. Moreover, the conformational fingerprint of prostate ACE was shown to be quite different from that for lung ACE [23] that opens up a theoretical possibility for the use the appearance of prostate-specific ACE in the blood as an early marker of prostate cancer. The difference in ACE concentrations in seminal fluid and plasma is just 50 [17, 18] - in contrast to >1 000 000 for PSA [26], therefore, we may expect that the frequency of false positive results with prostate ACE determination in the blood of patients with PC would be much lower than with quantification of PSA in the blood.

Here, we report the ACE phenotyping on prostate cancer tissues in comparison with normal prostate tissues and prostate tissues from patients with BPH. The data show that the catalytic parameter, namely, the ratio of the rates of the hydrolysis of two substrates hydrolyzed by the two active centers of ACE with different efficiency, ZPHL/HHL ratio [27], and the ratio of immunoreactive ACE protein to ACE activity have been significantly increased in patients with PC. Further, the conformational fingerprint of prostate ACE from patients with both BPH and PC differed noticeably from that from normal tissues. Therefore, ACE phenotyping of prostate biopsies could be an effective approach for an early diagnostics of prostate cancer and for differential diagnostics of BPH and PC.

## RESULTS AND DISCUSSION

### ACE activity in surgically removed prostate cancer tissues

In the first series of experiments, we performed ACE phenotyping in homogenates (prepared at 1:9 weight/volume ratio) of prostate tissues obtained after surgical removal of these tissues from 4 patients with PC and prostate tissues obtained by Trans-URethral Prostatectomy (TURP) of 6 patients with BPH (Supplementary Table 1). As a control, we used homogenates of postmortem prostate tissues from 2 subjects who died due to accidents.

**Figure 1:**
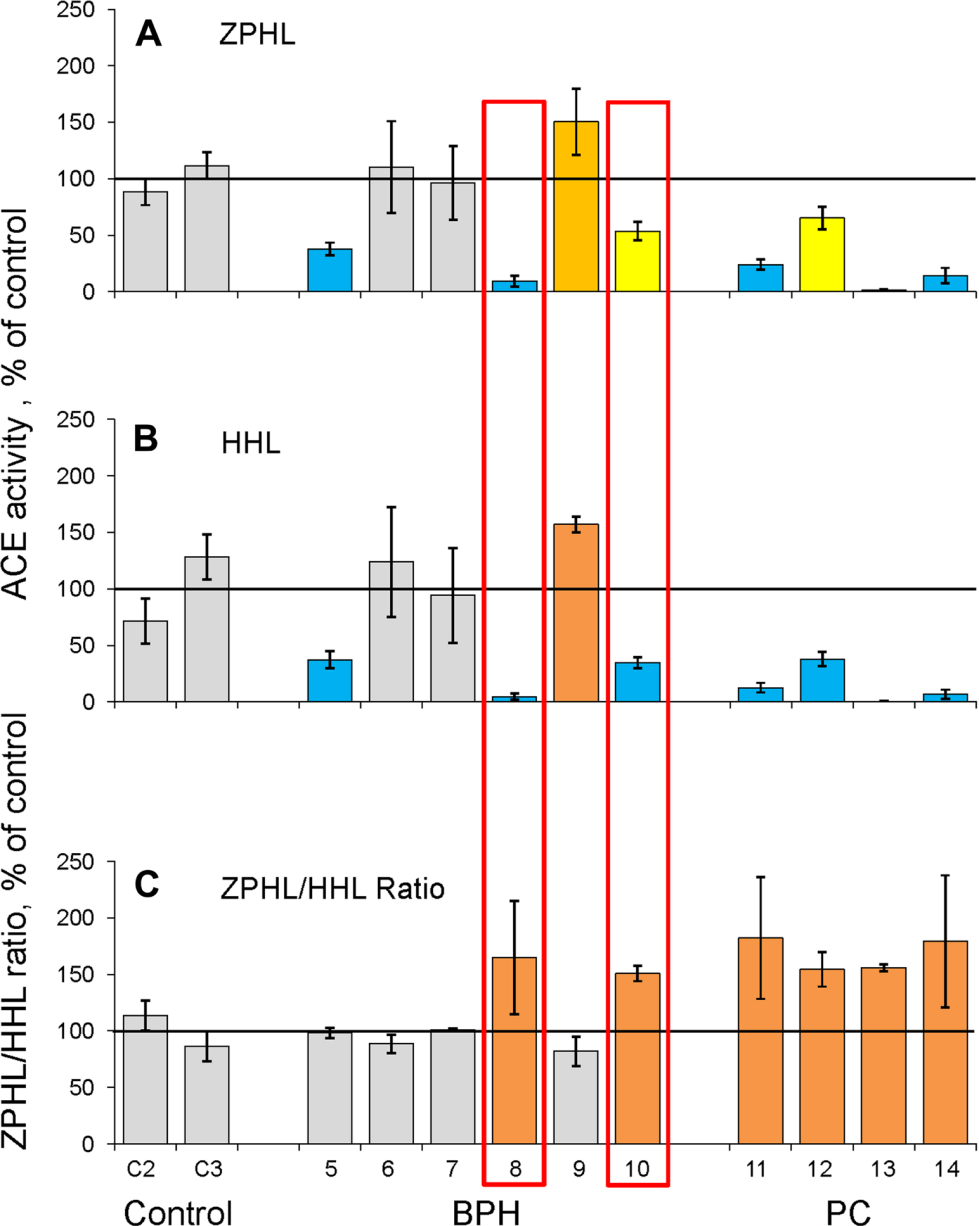
ACE activity in homogenates of prostate tissues (I cohort). Homogenates (1:9 weight/volume ratio) were prepared from surgically removed prostate tissues of 4 patients with PC, from TURP-treated prostate tissues of 6 patients with BPH and from tissues of 2 unrelated patients (died due to accident) used as controls. (**A**–**B**) ACE activity was quantified using a spectrofluorometric assay with ZPHL (2 mM, A) and HHL (5 mM, B) as substrates calculated as mU/mg of protein and expressed as a % from mean value for control samples. (**C**) ZPHL/HHL ratio. Data for ACE activity were expressed as ratios of the rates of the hydrolysis of Z-Phe-His-Leu to rates of the hydrolysis of Hip-His-Leu for each sample and presented as a % from mean value for control samples. Values increased more than 20% were highlighted with orange; more than 50% with brown. Bars highlighted with yellow - values decreased more than 20%, with blue - more than 50%. Bars were highlighted if values were statistically significant (^*^ *p* < 0.05). Data presented as a mean of at least 2 independent experiments in duplicates (with intra-assay standard deviations - SD <10%).

ACE activity in prostate cancer tissue homogenates (with ZPHL and HHL as substrates) was remarkably lower than in prostate tissues of unrelated patients (control) (Figure 1A and 1B), which is consistent with previously reported studies [19, 20]. However, ACE activity in prostate homogenates from patients with BPH (Figure 1A and 1B) was not elevated several fold as reported earlier [19, 20, 28]. We suggested that partial coagulation of prostate tissues during TURP at BPH in our case could lead to partial denaturation of ACE and, therefore, underestimation of real ACE concentration in prostate tissues of patients with BPH.

Because we measured ACE activity in prostate tissues with two substrates (ZPHL and HHL), we were able to calculate the ratio of the rates of their hydrolysis, ZPHL/HHL ratio. The two domains of ACE hydrolyze a range of natural and synthetic substrates, but with different efficacy [29–32]. The substrates ZPHL and HHL used for testing ACE activity in laboratories worldwide. The usual concentrations for these substrates are 2 mM for ZPHL and 5 mM for HHL, at pH 8.3. ACE domains hydrolyze these substrates with different rates. HHL is hydrolyzed faster (9-fold) by the C domain [29] in these conditions, whereas ZPHL hydrolyzed at similar rates by both domains [33]. As a result, the ratio of the rates of hydrolysis of these two substrates (ZPHL/HHL ratio) serves as a characteristic of a definite ACE form: for somatic two-domain human ACE it is about 1-1.5, for N domain – 5-7, and C domain – 0.6-0.8 [27]. The ZPHL/HHL ratio used primarily to detect the presence of common ACE inhibitors taken as a drug in patients’ blood at the time of blood sampling [27, 34, 35]. This parameter can also help to detect inactivation or inhibition of a separate domain, as the increase of this ratio can indicate inactivation/inhibition of the C domain, while the decrease of this ratio may be an indicator for inactivation/inhibition of N domain [27].

The ZPHL/HHL ratio is rather uniform parameter for native ACE in plasma or tissue homogenates and is characterized by very low inter-individual variability: while ACE activity determined with a single substrate in normal population varies 3-4 fold with standard deviation (SD) about ~30% [36, 37], SD for ZPHL/HHL ratio is only about 3–5% [27, 22]. All four prostate tissues of cancer patients were characterized by a significantly increased ZPHL/HHL ratio (Figure 1C, Supplementary Figure 1), while this parameter was not considerably increased in prostate tissue homogenates from patients with BPH taken a group (Supplementary Figure 1). However, individual approach revealed two homogenates (out of 6) of prostate tissues from patients with BPH, which were characterized by significantly increased ZPHL/HHL ratio and these very homogenates also demonstrated significantly decreased ACE activity (boxed in red in Figure 1). We could not exclude the possibility that decreasing of ACE activity and increasing ZPHL/HHL preceded the changes seen in histological slices and thus, could be used as an early indicator of tumor formation in enlarge prostate mass of BPH. Unfortunately, we could not reach again the patient ## 1-8 and 1-10 and to estimate their status presence regarding their prostate health.

We have found recently, that similar increase in ZPHL/HHL ratio was observed in 2 (out of 5) lung tissues from patients with lung cancer (unpublished observation). The increase in this parameter could reflect conformational changes in cancer ACE in these patients (see below), or, alternatively, the presence of tumor marker which expression is increased dramatically in tumor tissues (for example serum amyloid A protein [38]), which could bind to ACE and change its catalytic properties. We discovered recently (but not identified yet) similar ACE effector, which is present at high levels in normal spleen, but disappears in Gaucher spleen [25].

### ACE phenotyping on needle biopsy specimens

Despite the fact that these findings are novel and theoretically interesting, its diagnostic value is limited because it performed on prostate tissues after surgery or TURP. Therefore, we performed ACE phenotyping using prostate tissues obtained during prostate biopsy – standard diagnostic procedure to obtain prostate tissue for differential histological diagnosis between BPH and PC.

However, when we obtained needle biopsy specimens, we realized that very small amount of prostate issues that we obtained from each patient (between 3 and 10 mg) does not allow us to perform accurate prostate tissue homogenate preparation using Potter-Elveheim tissue homogenizer. Therefore, we had to combine prostate tissue biopsy specimens from each group for quantitative prostate tissue homogenate preparation and further ACE phenotyping. For the 2nd cohort, 4 postmortem prostate tissues from unrelated individuals were used as a control; BPH group consisted of 7 combined specimens while PC group was from 7 specimens (Supplementary Table 1). Despite the fact that the amount of tissues used for homogenate preparation after biopsy was 5-fold less than with surgically removed prostate tissues, we confirmed that at least ACE activity was significantly decreased only in homogenate from PC prostates and ZPHL/HHL ratio was also increased only in this homogenate (Supplementary Figure 2). ACE activity in prostate tissues of patients with BPH was higher than for control group in accordance with previous data [19, 20, 28] thus confirming our suggestion, that denaturation of ACE can occur during TURP (Supplementary Figure 1).

**Figure 2:**
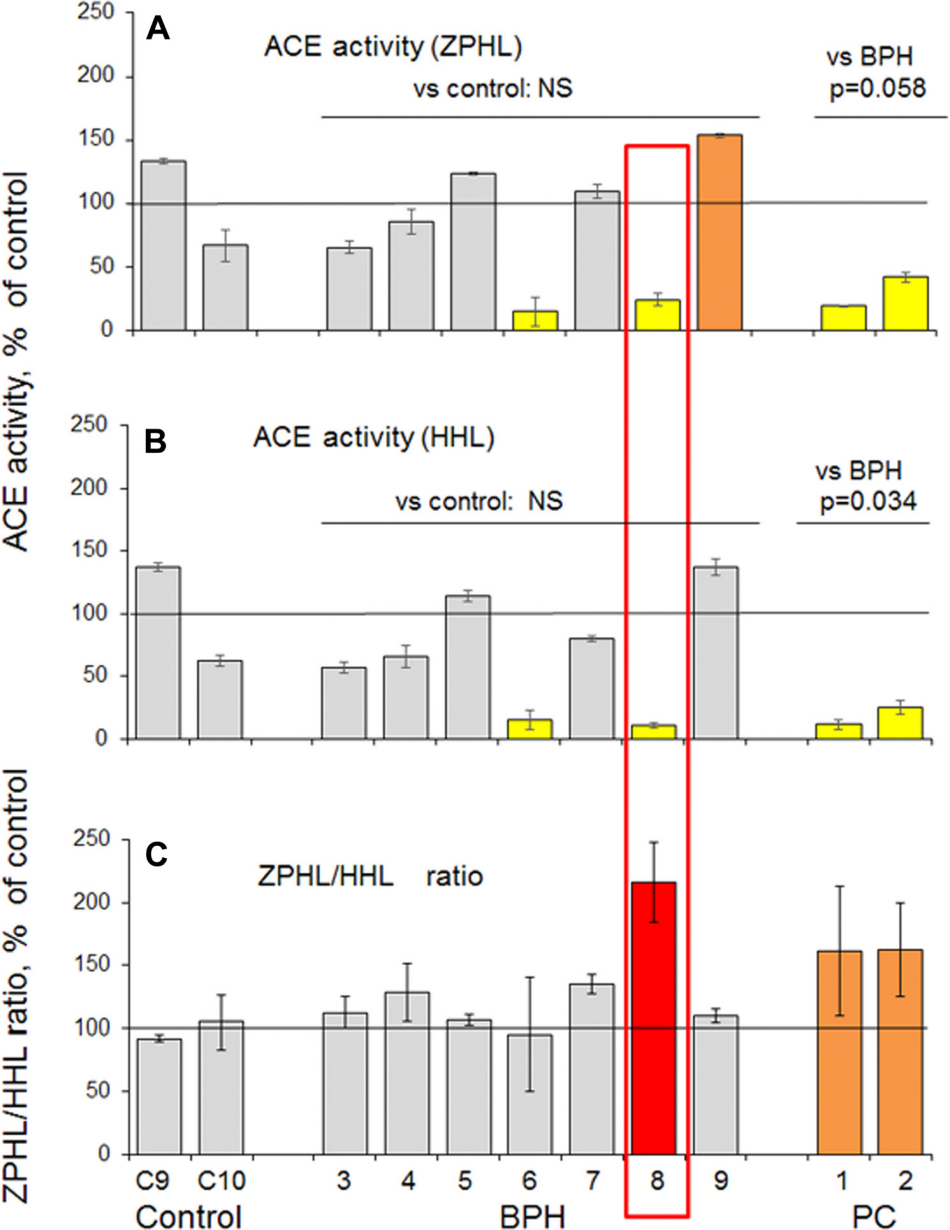
ACE activity in prostate tissues taken by biopsy (III cohort). (**A**–**B**) Prostate tissue homogenates were prepared from needle biopsies separately from 7 patients with BPH and 2 patients with PC. Homogenates from postmortem prostate tissue from 2 unrelated individuals (died due to accidents) served as controls. ACE activity was quantified as in Figure 1 legend and expressed as a % from mean values for control samples. (**C**) ZPHL/HHL ratio. Data presented as a mean of at least 2 independent experiments in du-or triplicates (with intra-assay standard deviations - SD <10%). Coloring was as in Figure 1, and individual values increased more than 100% were highlighted with red. Bars were highlighted if values were statistically significant (^*^ *p* < 0.05) from control values. Values in ACE activity for PC (as a group) were significantly lower than for BPH group (*p* = 0.058 for ZPHL and *p* = 0.034 for HHL).

In the next series, we used another tissue homogenizer – Speed Mill Plus (Analytik Jena, Jena, Germany), which was suitable for quantitative tissue homogenate preparation (using steel spheres) even from very small amount of tissue down to 3 mg, i. e. 30-fold less than in the case of surgically removed prostate tissues. This 3rd cohort, contained 2 postmortem prostate tissues as controls, 7 prostate tissues from patients with histologically proven BPH and two PC tissues (Supplementary Table 1). We again obtained similar results, namely, significant decrease of ACE activity and significant increase in ZPHL/HHL ratio for ACE in PC tissue homogenates (Figure 2). Moreover, using very small amount of tissue from needle biopsy we were able to identify patient (#3-8) from this cohort, boxed in red in Figure 2) which histologically (and clinically) was assigned to BPH group, whereas based on ACE phenotyping (significantly decreased ACE activity and increased ZPHL/HHP ratio) could represent rather PC group. (Similar to two patients ##1-8 and 1-10 from the 1^st^ cohort, Figure 1).

Repeated histological and clinical examination of patient #3-8 from the 3rd cohort demonstrated severe chronic prostatitis. Prostatic stroma was infiltrated predominantly by lymphocytes with the presence of sporadic histiocytes and neutrophils, while on separate sites destruction of prostatic epithelium was observed. There were no similar inflammatory changes in samples from other patients.

This finding (and especially using biopsy specimens, obtained during routine diagnostic procedure) strengthened our hypothesis that the changes in biochemical properties, namely, the decrease of ACE activity in tissue homogenates and increase of ZPHL/HHL ratio, could precede the changes seen on the cell level in histological slices and, thus, could be used as an early diagnostic of tumor formation in enlarge prostate mass of benign prostate hyperplasia using routinely taken biopsy specimens.

### Conformational fingerprinting of ACE in prostate tissues

In order to characterize the conformation of ACE in prostate tissues we performed conformational fingerprinting of ACE using a panel of mAbs directed against different epitopes located on the N and C domains of catalytically active human ACE [21]. We demonstrated previously that the pattern of precipitation of ACE activity by this set of mAbs provides a sensitive tool for detecting changes in local topography of the surface of ACE globule due to denaturation, inhibition [21, 22], mutations [39 and references therein, 40], or cell/tissue origin [21, 23, 25, 41].

**Figure 3:**
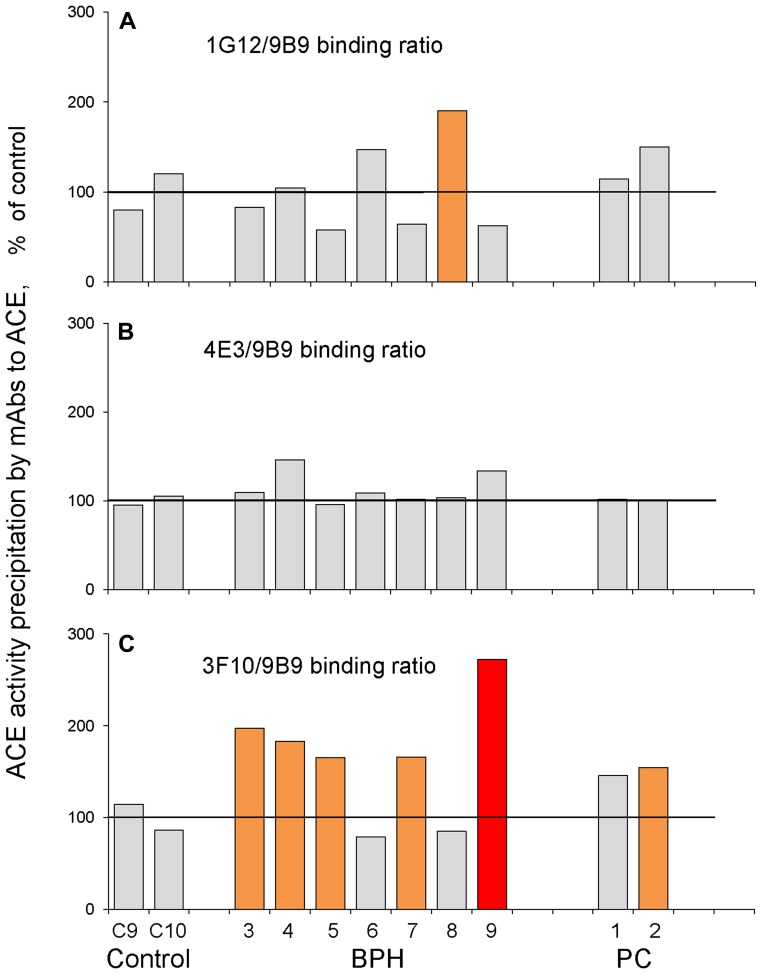
ACE conformational fingerprinting in prostate tissues (III cohort). ACE activity was precipitated by 4 different mAbs from homogenates from individual prostate biopsies from 2 PC patient, 7 BPH patients and 2 from postmortem counterparts. Data were presented as ratio of ACE activity precipitated by one of the tested mAbs to that precipitated by strongest mAb, 9B9. (**A**) 1G12/9B9: (**B**) 4E3/9B9, (**C**) 3F10/9B9. Data presented as a mean of at least 2 independent experiments in du-triplicates. Bars were highlighted if values were statistically significant (^*^ *p* < 0.05). Coloring is as in Figure 1.

Putative local conformational changes of ACE due to disease (defined as ratio of ACE activity precipitated by 6 different mAbs to the epitopes on the N and C domains to that by the most strong mAb 9B9) were not detected with 4 tested mAbs (3A5, i2H5, 5F1, 4E3) and were rather weak with mAb 1G12 for both PC and BPH prostate homogenates. However, these changes were very prominent with mAb 3F10, especially for BPH prostate tissue (Figure 3).

ACE phenotype parameters combined for each group from this 3rd cohort are presented on Supplementary Figure 3. Interestingly, when we calculated the relative ACE activity per amount of immunoreactive protein (detected with 4 mAbs) we found that this value was decreased for BPH group and further decreased for PC group (Supplementary Figure 3E). This finding indicates the presence of some specific ACE inhibitors in prostate tissue at pathology, especially in the prostate tumor tissues.

### Confirmation of ACE phenotype changes in two more cohorts

In order to confirm diagnostic value of the ACE phenotyping for the differential diagnostics of PC and BPH, we repeated ACE phenotyping on biopsy specimens collected by independent clinicians in another hospital. Histology on these specimens were performed later than ACE phenotyping, therefore, thus initially there were just two groups – normal tissues and prostate tissues from patients in question that were separated to two cohorts, 4rd and 5th. This is why the numbers of patients with PC and BPH were not equalized. As a result, ACE phenotyping in the 4th cohort was performed on 1 patient that later was assigned as PC and 5 patients with BPH. Six postmortem prostate tissues from patients died due to accidents were used as a control (Supplementary Figure 4). In the 5th cohort, ACE phenotyping was performed on 3 patients which later were assigned as PC and 6 patients with BPH with 3 controls (Supplementary Figure 5). Again, ACE activity was proven to be significantly decreased, while ZPHL/HHL ratio significantly increased, in prostate homogenates from patients with PC (Supplementary Figures 4 and 5). Again, we found one patient (# 4-4) in BPH group from 4th cohort (Supplementary Figure 4) and one patient (#5-1) from 5th cohort (Supplementary Figure 5) for which ZPHL/HHL ratio was increased as for patients with PC. Again, the majority of BPH samples in both cohorts demonstrated an increased 3F10/9B9 ratio (Supplementary Figures 4 and 5).

**Figure 4:**
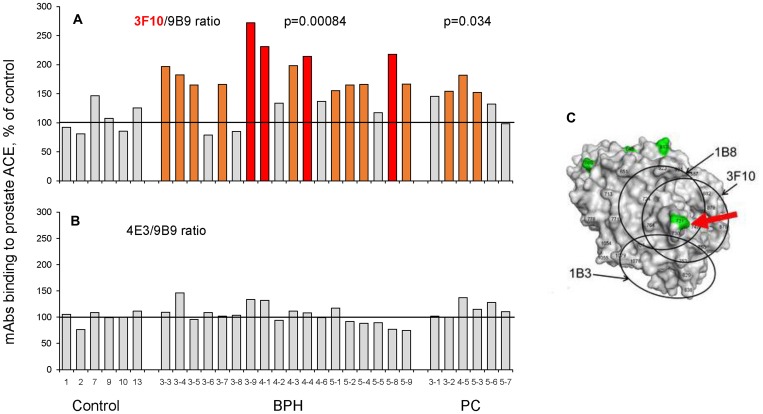
ACE conformational fingerprinting in biopsies from prostate tissues. ACE activity was precipitated by 3 different mAbs from homogenates from individual prostate biopsies: 6 of prostate cancer tissues, 18prostate tissues from patients with BPH and 6 from postmortem counterparts. Immunoprecipitated ACE activity is presented as ratio of ACE activity precipitated by one of the tested mAbs mAbs to that precipitated by mAb 9B9. (**A**) 3F10/9B9: (**B**) 4E3/9B9. (**C**) The structure of the C domain of ACE with marked potential glycosylation sites (by green) and epitopes for mAbs (by circles). Human C domain structure was based on PDB 1O86. The epitopes for mAbs were marked according to [42]. The positions of the epitopes are shown by circles. The potential sites of N-glycosylation are marked by green. Some amino acid residues are shown by numbers according to [11] for orientation. Data presented as a mean of at least 2 independent experiments. Bars were highlighted if values were statistically significant (^*^ *p* < 0.05). Coloring – as in Figure 1.

Figure 4 shows the binding of two mAbs, 3F10 and 4E3 to prostate ACE from all needle biopsies, normalized for binding of mAb 9B9. While the 4E3/9B9 ratio did not change for ACE from BPH and PC tissues, the majority of BPH and PC samples demonstrated statistically increased 3F10/9B9 ratio (169%, *p* = 0.0084, and 144%, *p* = 0.0034, correspondingly) which makes this parameter a new marker for BPH development. Epitope for mAb 3F10 is located on the C domain of ACE and includes potential glycosylation site Asn731 [42, 23]. We hypothesized that an increase in 3F10/9B9 ratio may result from different structure of glycan in this potential glycosylation site, likely greater sialylation, in BPH and tumor ACE.

Increased sialylation during cancerous transformation and tumor progression is well documented [43–45] and most likely results from the overexpression of sialyltransferases [45, 46] helping, therefore, tumor cells to withstand apoptosis. Cell and tissue specificity of these enzymes suggests that each type (including probably malignant cells) have a unique “sialome” which may be used to document cell origin or pathology [47]. Our hypothesis, that observed changes in binding of mAb 3F10 to ACE from BPH and PC prostate tissues (Figure 4) are due to hypersialylation of ACE molecules is supported by the finding that this very mAb bind better to plasma ACE than to lung ACE (Figure 10A in [35], and plasma ACE is known to be more sialylated than its “parent” lung enzyme as a result of elimination of less sialylated proteins from the blood by liver lectins [48].

**Figure 5:**
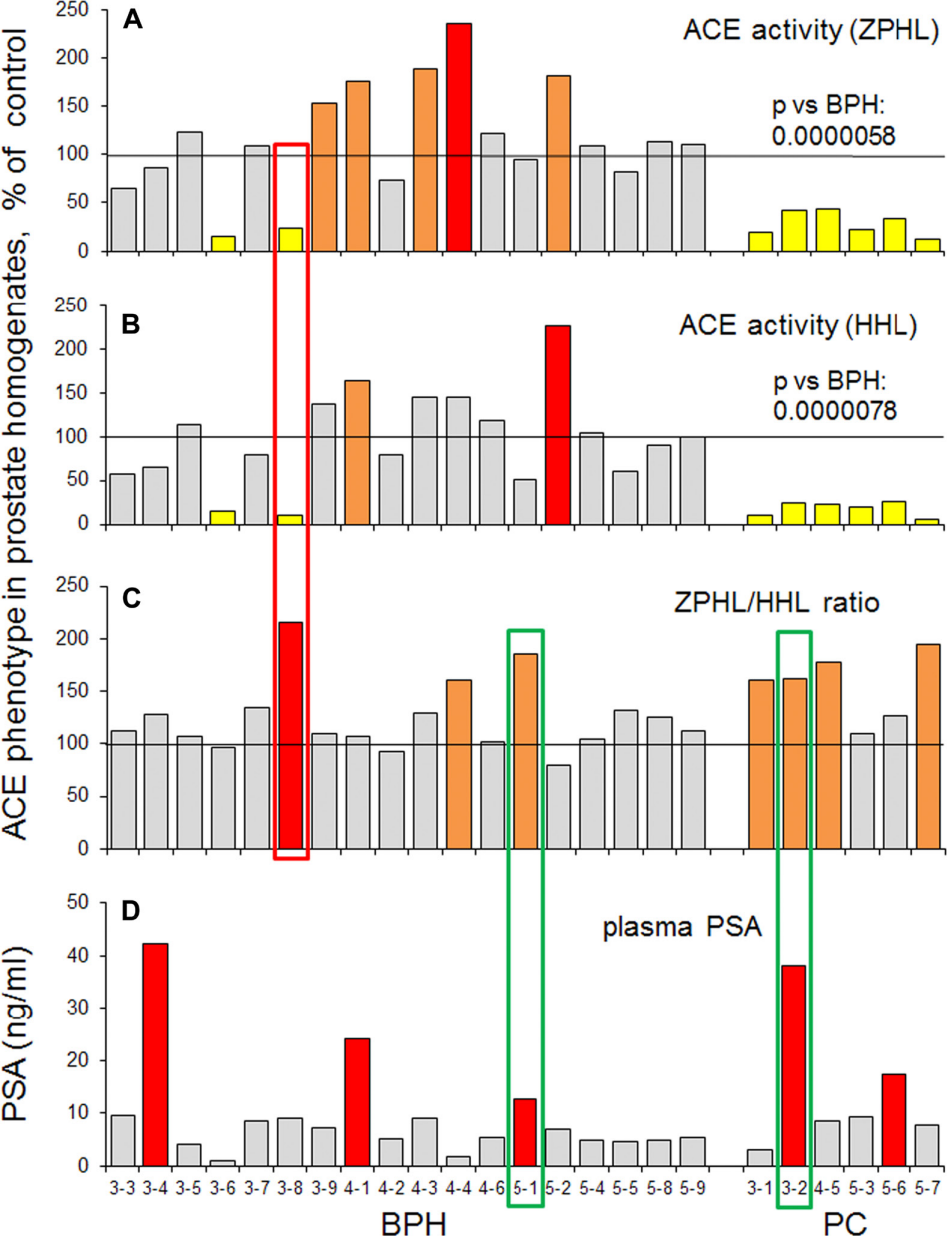
ACE activity in biopsies of prostate tissues. Data on ACE activity in individual samples were presented for all biopsies that were analysed separately (cohorts III, IV and V): 6 samples of prostate cancer tissues, 18 prostate tissues from patients with BPH and 6 from postmortem counterparts. ACE activity was expressed as a % from mean value for control samples. (**A**) with ZPHL as a substrate; (**B**) with HHL as a substrate; (**C**) as ZPHL/HHL ratio. (**D**) the level of PSA in individual homogenates. Coloring as in Figure 1. Bars were highlighted if values were statistically significant (^*^ *p* < 0.05). Data presented as a mean of at least 2 independent experiments in du-or triplicates (with intra-assay standard deviations - SD <10%). Values in ACE activity for PC (as a group) were significantly lower than for BPH group (*p* = 0.0000058 for ZPHL and *p* = 0.0000078 for HHL).

We combined together several parameters of prostate ACE phenotype for all needle biopsies, 18 biopsies from patients with BPH and 6 patients with PC (based on histology), shown in Figure 5. All ACE-related parameters for patients with PC look very homogenous. One patient (#3-8, boxed with red) with histology-based BPH could be considered as PC-like by virtue of the decreased ACE activity and increased ZPHL ratio. Comparison of ACE-related parameters (Figure 5A–5C) with PSA in the blood (Figure 5D) showed that two patients demonstrated increased ZPHL/HHL ratio and PSA, one patient, #5-1, from BPH group and another, #3-2, from PC group (boxed with green).

**Figure 6:**
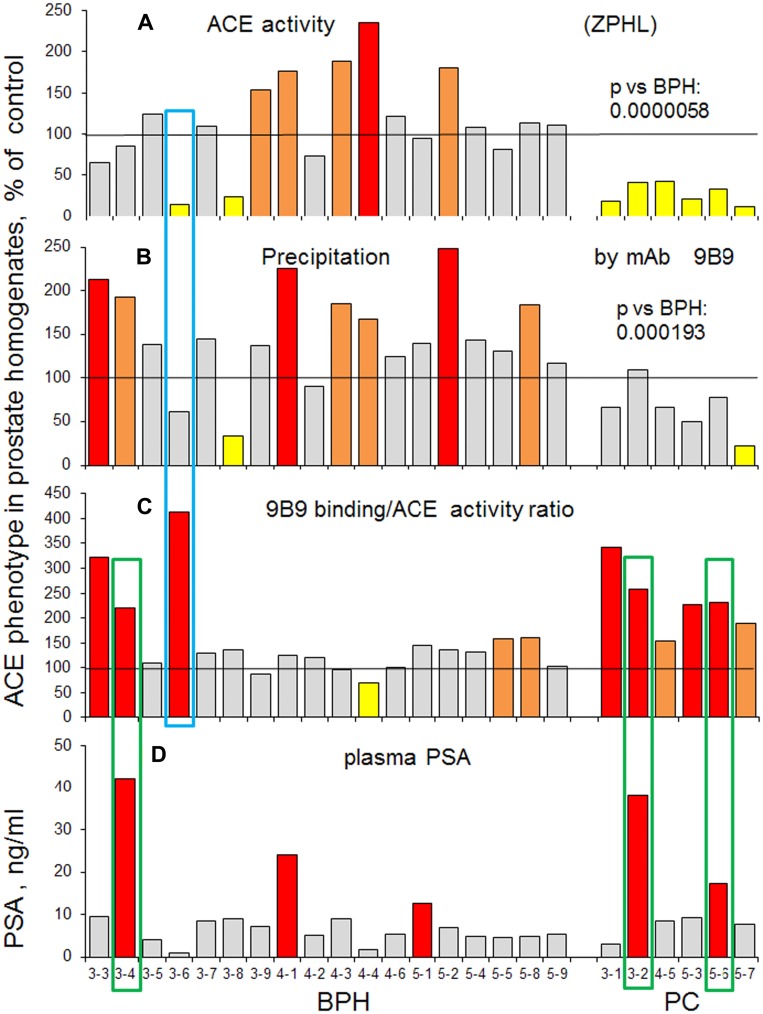
Prostate ACE phenotype in BPH and PC. Individual data on ACE phenotype characteristics were presented for all biopsies that were analysed separately as in Figure 5 and expressed as a % from mean value for control samples. (**A**) ACE activity with ZPHL (the same as Figure 5A). (**B**) amount of immunoreactive ACE protein (determined by precipitation with mAb 9B9. Values in ACE activity precipitation by mAb 9B9 for PC (as a group) were significantly lower than for BPH group (*p* = 0.000193). (**C**) the ratio of immunoreactive ACE protein to ACE activity. (**D**) PSA in the blood. Coloring as in Figure 1. Data presented as a mean of at least 2 independent experiments in du-or triplicates (with intra-assay standard deviations - SD <10%).

Figure 6 shows a comparison of ACE activity (Figure 6A) and the amount of immunoreactive ACE protein precipitated by mAb 9B9 (Figure 6B). We introduced a novel parameter for ACE phenotyping, ratio of immunoreactive ACE protein to ACE activity (Figure 6C). Interestingly, this parameter was increased dramatically (233.7% ± 64.9, *p* = 0.001) for ACE from all patients with PC in comparison to controls (Figure 6C), while not all these patients exhibited increased PSA (Figure 6D). One patient with BPH (#3-6) looks as patient with PC, based on the decreased ACE activity and increased ACE immunoreactive ACE protein/ACE activity ratio (boxed with blue). Also, there were two patients with simultaneously increased PSA and 9B9/ACE activity ratio in PC group and one such patient in BPH group (boxed with green). Therefore, it seems that ACE-related new parameters (at least for prostate tissue ACE) better correspond to PC histology than PSA in the blood.

In conclusion, we performed ACE phenotyping of patient’s prostate cancer tissue versus prostate tissues from patients with BPH and found that ACE activity, kinetic characteristic and surface conformation of ACE in prostate cancer tissue homogenates differ from ACE in prostate homogenates of patients with BPH, as well as controls. Moreover, we further demonstrated that the surface conformation of ACE is also disease-specific. We suggest that ACE phenotyping of prostate biopsies has a potential in a future to be an effective approach for an early diagnostics of prostate cancer and for differential diagnostics of BPH and PC.

These findings may be clinically relevant especially with the recognition that tissue renin-angiotensin system is a target not only for therapy of cardiovascular disorders [49] but also in cancer chemotherapy [15].

## MATERIALS AND METHODS

### Study participants

This pilot study was conducted according to the principles of the Helsinki Declaration and was approved by the local ethics committees of Medical Center of Moscow State University and Lopatkin Research Institute of Urology and Interventional Radiology. All tissue procurement procedures were carried out in accordance with institutional guidelines. After obtaining a written informed consent from the patients or next of kin, surgical specimens of prostate cancer or benign prostate hyperplasia, as well as post mortem prostate and lung tissues without pathology from individuals died from accident, were collected for the measurements of enzymatic activity and immunochemical characterization. Patient selection is outlined in Supplementary Table 1. ACE phenotyping was performed on 5 sets of prostate tissues from 31 patients with BPH (median age – 66.2 years), 17 patients with PC (median age – 68.5 years) and 8 post mortem prostate tissues from unrelated individuals (median age –32.7 years) that were used as a controls.

### Preparation of prostate tissue homogenates

In the first series of experiments, we prepared prostate (and lung) tissue homogenates by homogenization of 100 mg of tissue in 900 μl of PBS using Potter-Elveheim homogenizer. In all other series we used needle biopsies, where the amount of tissue was minimal (from 3 to 12 mg), which were used for the preparation of homogenates in 1 ml of PBS using Speed Mill Plus (Analytik Jena, Jena, Germany) with steel spheres. After homogenization, Triton X-100 was added to the final concentration 0.25% for the extraction of membrane-bound ACE.

### Prostate needle biopsies

Thirty eight patients (belonging to 4 separate cohorts, see Supplementary Table 1) were scheduled for ultrasound-guided prostate biopsy. In concordance to the European guidelines for detection of PC, indication for biopsy was either elevated PSA levels (≥ 4 ng/ml) or suspicious digital rectal examination [50]. Patients with proven PC in history were not included. Transrectal ultrasound was performed on an Aixplorer (SuperSonic Imagine, Aix-en-Provence, France) with an SE12-3 MHz probe including grey-scale ultrasound, color Doppler, and shear wave elastography (SWE) imaging

Nodules and other suspicious areas were noted by the radiologist during grey-scale examination. Shear wave elastography is based on the fact that the stiffness of the cancerous tissue in the prostate is higher than the stiffness of the benign tissue. SWE provides a dynamic quantitative map of soft tissue visco-elastic properties in real-time. The stiffness of tissue is color-coded for each pixel and displayed as an overlay on the grey-scale image. A stiffness value greater than 35 kPa is suggestive of a malignancy [51]. Recent studies demonstrated the ability of SWE to detect prostate cancer nodules with good accuracy [52, 53].

After performance of grey-scale ultrasound and SWE, systematic and targeted biopsies were taken in up to gray scale ultrasound or SWE-suspicious areas. All biopsies were performed with US guidance by using a disposable 18-gauge prostate needle (Tru-Cut needle, Bard, Covington, Ga). The systematic biopsy protocol included 36 peripheral posterior biopsies, with paramedian and lateral cores for each sextant from the apex, midgland and base of the prostate. 1–2 cores were sampled from hypoechogenic areas in gray scale ultrasound or from SWE-suspicious areas, followed by 36 systematic biopsies. Then three additional cores were taken from each suspicious area for subsequent measurements of enzymatic activity and immunochemical characterization ACE in this area of prostate tissue. According to the results of the histological study, the morphological characteristics of these suspicious areas were analyzed and compared with the activity and immunochemical characterization of ACE.

### Histology

Histology was performed according to standard techniques. Samples were fixed in 4% neutral buffered formaldehyde for 48 hours and embedded in paraffin. We performed hematoxylin and eosin (H&E) staining. Prepared sections were studied microscopically using LeicaDMLB2 light microscope by two independent observers.

### Prostate tissue homogenates

For surgically removed prostate tissues homogenization was performed in PBS (pH 7.5) (1:9 weight/volume ratio) using Potter-Elveheim glass or mechanical tissue homogenizer. Then Triton X-100 was added to final concentration of 0.25% for overnight incubation and after centrifugation supernatant was used as source of prostate ACE.

### ACE activity assay

ACE activity in prostate and lung tissue homogenates was measured using a fluorimetric assay with two ACE substrates, 2 mM Z-Phe-His-Leu or 5 mM Hip-His-Leu [37]. Briefly, 20–40 μl aliquots of samples were added to 200 μl of ACE substrate and incubated for the appropriate time at 37° C. His-Leu product was quantified based on its complex forming with *o*-phtaldialdehyde.

### Immunological characterization of ACE (enzyme-captured immunoprecipitation assay)

Ninety six-well plates (Corning, Corning, NY) were coated with 16 anti-ACE mAbs using precoated goat anti-mouse IgG (Pierce, Rockford, IL) as a capture and incubated with tissue homogenates containing ACE. Plate-bound ACE activity was measured using ACE substrate Z-Phe-His-Leu directly in the wells [33, 21].

### Statistical analysis

All data are presented as mean ± SD. Significance was calculated using the Mann-Whitney test with STATISTICA 6 (StatSoft, Inc., OK).

## SUPPLEMENTARY MATERIALS





## Abbreviations

ACE: angiotensin-converting enzyme
mAbs: monoclonal antibodies
ZPHL: carbobenzoxy-L-phenylalanyl-L-histidyl-L-leucine
HHL: hippuryl-L-histidyl-L-leucine
PBS: phosphate buffered saline

## References

[1] Ferlay J, Soerjomataram I, Ervik M, Dikshit R, Eser S, Mathers C GLOBOCAN 2013 v.1.0. Cancer Incidence and mortality worldwide. IARCCancerBase #11. https://gco.iarc.fr/.

[2] Chatterjee SK, Zetter BR Cancer biomarkers: knowing the present and predicting the future. Future Oncol. 2005; 1:37–50. 10.1517/14796694.1.1.37. 16555974

[3] Murphy DG, Ahlering T, Catalona WJ, Crowe H, Crowe J, Clarke N, Cooperberg M, Gillatt D, Gleave M, Loeb S, Roobol M, Sartor O, Pickles T, et al The Melbourne consensus statement on the early detection of prostate cancer. BJU Int. 2014; 113:186–188. 10.1111/bju.12556. 24206066

[4] Ahmed HU Prostate cancer: melbourne consensus-noble but misguided. Nat Rev Urol. 2014; 11:250–251. 10.1038/nrurol.2014.65. 24662731

[5] Park SC, Shin YS, Zhang LT, Kim DS, Kim SZ, Park NC, Ahn TY, Kim JJ, Lee SW, So I, Park JK Prospective investigation of change in the prostate-specific antigens after various urological procedures. Clin Interv Aging. 2015; 10:1213–1218. 10.2147/CIA.S84570. 26251583 PMC4524270

[6] Catalona WJ, Partin AW, Slawin KM, Brawer MK, Flanigan RC, Patel A, Richie JP, de Kernion JB, Walsh PC, Scardino PT, Lange PH, Subong EN, Parson RE, et al Use of the percentage of free prostate-specific antigen to enhance differentiation of prostate cancer from benign prostatic disease: a prospective multicenter clinical trial. JAMA. 1998; 279:1542–1547. 10.1001/jama.279.19.1542. 9605898

[7] Roddam AW, Duffy MJ, Hamdy FC, Ward AM, Patnick J, Price CP, Rimmer J, Sturgeon C, White P, Allen NE; NHS Prostate Cancer Risk Management Programme Use of prostate-specific antigen (PSA) isoforms for the detection of prostate cancer in men with a PSA level of 2–10 ng/ml: systematic review and meta-analysis. Eur Urol. 2005; 48:386–399. 10.1016/j.eururo.2005.04.015. 15982797

[8] Etzioni R, Penson DF, Legler JM, di Tommaso D, Boer R, Gann PH, Feuer EJ Overdiagnosis due to prostate-specific antigen screening: lessons from U.S. prostate cancer incidence trends. J Natl Cancer Inst. 2002; 94:981–990. 10.1093/jnci/94.13.981. 12096083

[9] Fillela X, Foj L Emerging biomarkers in the detection and prognosis of prostate cancer. Clin Chem Lab Med. 2015; 53:963–973. 10.1515/cclm-2014-0988. 25581761

[10] Leyten GH, Hessels D, Smit FP, Jannink SA, de Jong H, Melchers WJ, Cornel EB, de Reijke TM, Vergunst H, Kil P, Knipscheer BC, Hulsbergen-van de Kaa CA, Mulders PF, et al Identification of a Candidate Gene Panel for the Early Diagnosis of Prostate Cancer. Clin Cancer Res. 2015; 21:3061–3070. 10.1158/1078-0432.CCR-14-3334. 25788493

[11] Soubrier F, Alhenc-Gelas F, Hubert C, Allegrini J, John M, Tregear G, Corvol P Two putative active centers in human angiotensin I-converting enzyme revealed by molecular cloning. Proc Natl Acad Sci USA. 1988; 85:9386–9390. 10.1073/pnas.85.24.9386. 2849100 PMC282757

[12] Sturrock ED, Anthony S, Danilov SM Peptidyl-dipeptidase A/Angiotensin I-converting enzyme. In: Rawlings ND, and Salvesen G, eds Handbook of Proteolytic Enzymes. 3rd ed Oxford: Academic Press; 2012:480–494. 10.1016/B978-0-12-382219-2.00098-3.

[13] Bernstein K, Ong FS, Blackwell WL, Shah KH, Giani JF, Gonzalez-Villalobos RA, Shen XZ, Fuchs S, Touyz RM A modern understanding of the traditional and nontraditional biological functions of angiotensin-converting enzyme. Pharmacol Rev. 2012; 65:1–46. 10.1124/pr.112.006809. 23257181 PMC3565918

[14] Lever AF, Hole DJ, Gillis CR, McCallum IR, McInnes GT, MacKinnon PL, Meredith PA, Murray LS, Reid JL, Robertson JW Do inhibitors of angiotensin-I-converting enzyme protect against risk of cancer? Lancet. 1998; 352:179–84. 10.1016/S0140-6736(98)03228-0. 9683206

[15] Pinter M, Jain RK Targeting the renin-angiotensin system to improve cancer treatment: implication for immunotherapy. Sci Transl Med. 2017; 9:eaan5616. 10.1126/scitranslmed.aan5616. 28978752 PMC5928511

[16] Hohlbrugger G, Pschorr J, Dahlheim H Angiotensin I converting enzyme in the ejaculate of fertileand infertile men. Fertil Steril. 1984; 41:324–325. 10.1016/S0015-0282(16)47614-4. 6321247

[17] Krassnigg F, Niederhauser H, Fink E, Frick J, Schill WB Angiotensin converting enzyme inhuman seminal plasma is synthesized by the testis, epididymis and prostate. Int J Androl. 1989; 12:22–28. 10.1111/j.1365-2605.1989.tb01282.x. 2541085

[18] Nikolaeva MA, Balyasnikova IV, Alexinskaya MA, Metzger R, Franke FE, Albrecht RF 2nd, Kulakov VI, Sukhikh GT, Danilov SM Testicular isoform of angiotensin I-converting enzyme (ACE, CD143) on the surface of human spermatozoa: revelation and quantification using monoclonal antibodies. Am J Reprod Immunol. 2006; 55:54–68. 10.1111/j.1600-0897.2005.00326.x. 16364013

[19] Yokoyama M, Hiwada K, Kokubu T, Takaha M, Takeuchi M Angiotensin-converting enzyme in human prostate. Clin Chim Acta. 1980; 100:253–258. 10.1016/0009-8981(80)90274-0. 6153302

[20] van Sande M, Inokuchi J, Nagamatsu A, Scharpé S, Neels H, Van Camp K Tripeptidyl carboxypeptidase activity of angiotensin-converting enzyme in human tissues of the urogenital tract. Urol Int. 1985; 40:100–102. 10.1159/000281046. 2581348

[21] Danilov SM, Balyasnikova IV, Danilova AS, Naperova IA, Arablinskaya NE, Borisov SE, Metzger R, Franke FE, Schwartz DE, Gachok IV, Trakht IN, Kost OA, Garcia JG Conformational fingerprinting of the angiotensin-converting enzyme (ACE): Application in sarcoidosis. J Proteome Res. 2010; 9:5782–5793. 10.1021/pr100564r. 20873814

[22] Petrov MN, Shilo VY, Tarasov AV, Schwartz DE, Garcia JGN, Kost OA, Danilov SM Conformational changes of blood ACE in chronic uremia. PLoS One. 2012; 7:e49290. 10.1371/journal.pone.0049290. 23166630 PMC3500299

[23] Kryukova OV, Tikhomirova VE, Golukhova EZ, Evdokimov VV, Kalantarov GF, Trakht IN, Schwartz DE, Dull RO, Gusakov AV, Uporov IV, Kost OA, Danilov SM Tissue Specificity of human angiotensin I-converting enzyme. PLoS One. 2015; 10:e0143455. 10.1371/journal.pone.0143455. 26600189 PMC4658169

[24] Kost OA, Tikhomirova VE, Kryukova OV, Gusakov AV, Bulaeva NI, Evdokimov VV, Golukhova EZ, Danilov SM A conformational fingerprint of angiotensin-converting enzyme. Russ J Bioorganic Chem. 2018; 44:52–63. 10.1134/S1068162018010107.

[25] Danilov SM, Tikhomirova VE, Metzger R, Naperova IA, Bukina TM, Goker-Alpan O, Tayebi N, Gayfullin NM, Schwartz DE, Samokhodskaya LM, Kost OA, Sidransky E ACE phenotyping in Gaucher disease. Mol Genet Metab. 2018; 123:501–510. 10.1016/j.ymgme.2018.02.007. 29478818 PMC5891352

[26] Lovgren J, Valtonen-Andre C, Marsal K, Lilja H, Lundwall A Measurement of prostate-specific antigen and human glandular kallikrein 2 in different body fluids. J Androl. 1999; 20:348–355. 10386814

[27] Danilov SM, Balyasnikova IV, Albrecht RF 2nd, Kost OA Simultaneous determination of ACE activity with two substrates provides information on the status of somatic ACE and allows detection of inhibitors in human blood. J Cardiovasc Pharmacol. 2008; 52:90–103. 10.1097/FJC.0b013e31817fd3bc. 18645413

[28] Nassis L, Frauman AG, Ohishi M, Zhuo J, Casley DJ, Johnston CI, Fabiani ME Localization of angiotensin-converting enzyme in the human prostate: pathological expression in benign prostatic hyperplasia. J Pathol. 2001; 195:571–579. 10.1002/path.999. 11745693

[29] Wei L, Alhenc-Gelas F, Corvol P, Clauser E The two homologous domains of human angiotensin I-converting enzyme are both catalytically active. J Biol Chem. 1991; 266:9002–9008. 1851160

[30] Jaspard E, Wei L, Alhenc-Gelas F Differences in the properties and enzymatic specificities of the two active sites of angiotensin I-converting enzyme (kininase II). Studies with bradykinin and other natural peptides. J Biol Chem. 1993; 268:9496–9503. 7683654

[31] Georgiadis D, Beau F, Czarny B, Cotton J, Yiotakis A, Dive V Roles of the two active sites of somatic angiotensin-converting enzyme in the cleavage of angiotensin I and bradykinin: insights from selective inhibitors. Circ Res. 2003; 93:148–154. 10.1161/01.RES.0000081593.33848.FC. 12805239

[32] Skirgello OE, Binevski PV, Pozdnev VF, Kost OA Kinetic probes for inter-domain cooperation in human somatic angiotensin-converting enzyme. Biochem J. 2005; 391:641–647. 10.1042/BJ20050702. 16033330 PMC1276965

[33] Danilov S, Jaspard E, Churakova T, Towbin H, Savoie F, Wei L, Alhenc-Gelas F Structure-function analysis of angiotensin I-converting enzyme using monoclonal antibodies. Selective inhibition of the amino-terminal active site. J Biol Chem. 1994; 269:26806–26814. 7523412

[34] Danilov SM, Tovsky SI, Schwartz DE, Dull RO ACE phenotyping as a guide toward personalized therapy with ACE inhibitors. J Cardiovasc Pharmacol Ther. 2017; 22:374–386. 10.1177/1074248416686188. 28587581

[35] Danilov SM, Tikhomirova VE, Kryukova OV, Balatsky AV, Bulaeva NI, Golukhova EZ, Bokeria LA, Samokhodskaya LM, Kost OA Conformational fingerprint of blood and tissue ACEs: personalized approach. PLoS One. 2018; 13:e0209861. 10.1371/journal.pone.0209861. 30589901 PMC6307727

[36] Alhenc-Gelas F, Richard J, Courbon D, Warnet JM, Corvol P Distribution of plasma angiotensin I-converting enzyme levels in healthy men: Relationship to environmental and hormonal parameters. J Lab Clin Med. 1991; 117:33–39. 1846167

[37] Danilov S, Savoie F, Lenoir B, Jeunemaitre X, Azizi M, Tarnow L, Alhenc-Gelas F Development of enzyme-linked immunoassays for human angiotensin I converting enzyme suitable for large-scale studies. J Hypertens. 1996; 14:719–727. 10.1097/00004872-199606000-00007. 8793694

[38] Cho WC, Yip TT, Cheng WW, Au JS Serum amyloid A is elevated in the serum of lung cancer patients with poor prognosis. Br J Cancer. 2010; 102:1731–35. 10.1038/sj.bjc.6605700. 20502455 PMC2883701

[39] Danilov SM, Wade MS, Schwager SL, Douglas RG, Nesterovitch AB, Popova IA, Hogarth KD, Bhardwaj N, Schwartz DE, Sturrock ED, Garcia JG A novel angiotensin I-converting enzyme mutation (S333W) impairs N-domain enzymatic cleavage of the anti-fibrotic peptide, AcSDKP. PLoS One. 2014; 9:e88001. 10.1371/journal.pone.0088001. 24505347 PMC3913711

[40] Danilov SM, Lünsdorf H, Akinbi HT, Nesterovitch AB, Epshtein Y, Letsiou E, Kryukova OV, Piegeler T, Golukhova EZ, Schwartz DE, Dull RO, Minshall RD, Kost OA, et al Lysozyme and bilirubin bind to ACE and regulate its conformation and shedding. Sci Rep. 2016; 6:34913. 10.1038/srep34913. 27734897 PMC5062130

[41] Tikhomirova VE, Kost OA, Kryukova OV, Golukhova EZ, Bulaeva NI, Zholbaeva AZ, Bokeria LA, Garcia JGN, Danilov SM ACE phenotyping in human heart. PLoS One. 2017; 12:e0181976. 10.1371/journal.pone.0181976. 28771512 PMC5542439

[42] Naperova IA, Balyasnikova IV, Schwartz DE, Watermeyer J, Sturrock DE, Kost OA, Danilov SM Mapping of conformational mAb epitopes to the C domain of human angiotensin I-converting enzyme (ACE). J Proteome Res. 2008; 7:3396–3411. 10.1021/pr800142w. 18576678

[43] Bull C, Stoel MA, den Brok MH, Adema GJ Sialic acids sweeten a tumor’s life. Cancer Res. 2014; 74:3199–3204. 10.1158/0008-5472.CAN-14-0728. 24830719

[44] Stowell SR, Ju T, Cummings RD Protein glycosylation in cancer. Annu Rev Pathol. 2015; 10:473–510. 10.1146/annurev-pathol-012414-040438. 25621663 PMC4396820

[45] Schauer R, Kamerling JP Exploration of the sialic acid world. Adv Carbohydr Chem Biol. 2018; 75:1–213. 10.1016/bs.accb.2018.09.001. 30509400 PMC7112061

[46] Takashima S, Tsuji S Functional diversity of mammalian sialyltransferases. Trends Glycosci Glycotechnol. 2011; 23:178–193. 10.4052/tigg.23.178.

[47] Cohen M, Varki A The sialome – far more than the sum of its parts. OMICS. 2010; 14:455–464. 10.1089/omi.2009.0148. 20726801

[48] Ashwell G, Harford J Carbohydrate-specific receptors of the liver. Annu Rev Biochem. 1982; 51:531–554. 10.1146/annurev.bi.51.070182.002531. 6287920

[49] Bader M Tissue renin-angiotensin-aldosterone systems:targets for pharmacological therapy. Annu Rev Pharmacol Toxicol. 2010; 50:439–465. 10.1146/annurev.pharmtox.010909.105610. 20055710

[50] Mottet N, Bellmunt J, Bolla M, Briers E, Cumberbatch MG, De Santis M, Fossati N, Gross T, Henry AM, Joniau S, Lam TB, Mason MD, Matveev VB, et al EAU-ESTRO-SIOG Guidelines on prostate cancer. Part 1: Screening, diagnosis, and local treatment with curative intent. Eur Urol. 2017; 71:618–629. 10.1016/j.eururo.2016.08.003. 27568654

[51] Barr RG, Cosgrove D, Brock M, Cantisani V, Correas JM, Postema AW, Salomon G, Tsutsumi M, Xu HX, Dietrich CF WFUMB Guidelines and recommendations on the clinical use of ultrasound elastography: Part 5. Prostate. Ultrasound Med Biol. 2017; 43:27–48. 10.1016/j.ultrasmedbio.2016.06.020. 27567060

[52] Boehm K, Salomon G, Beyer B, Schiffmann J, Simonis K, Graefen M, Budaeus L Shear wave elastography for localization of prostate cancer lesions and assessment of elasticity thresholds: implications for targeted biopsies and active surveillance protocols. J Urol. 2015; 193:794–800. 10.1016/j.juro.2014.09.100. 25264337

[53] Correas JM, Tissier AM, Khairoune A, Vassiliu V, Méjean A, Hélénon O, Memo R, Barr RG Prostate cancer: Diagnostic performance of real-time shear-wave elastography. Radiology. 2015; 275:280–289. 10.1148/radiol.14140567. 25599156

