# Supplementary material for: Tissue ACE phenotyping in prostate cancer
Source: Oncotarget. 2019 Oct 29;10(59):6349–61. doi: 10.18632/oncotarget.27276 (PMC6824872; doi:10.18632/oncotarget.27276)
Supplement: Supplementary file 1 [file oncotarget-10-6349-s001.pdf]

Tissue ACE phenotyping in prostate cancer

SUPPLEMENTARY MATERIALS

Supplementary Table 1: The designation of patients in different cohorts and description of prostate tissues used for ACE phenotyping. See Supplementary Table 1

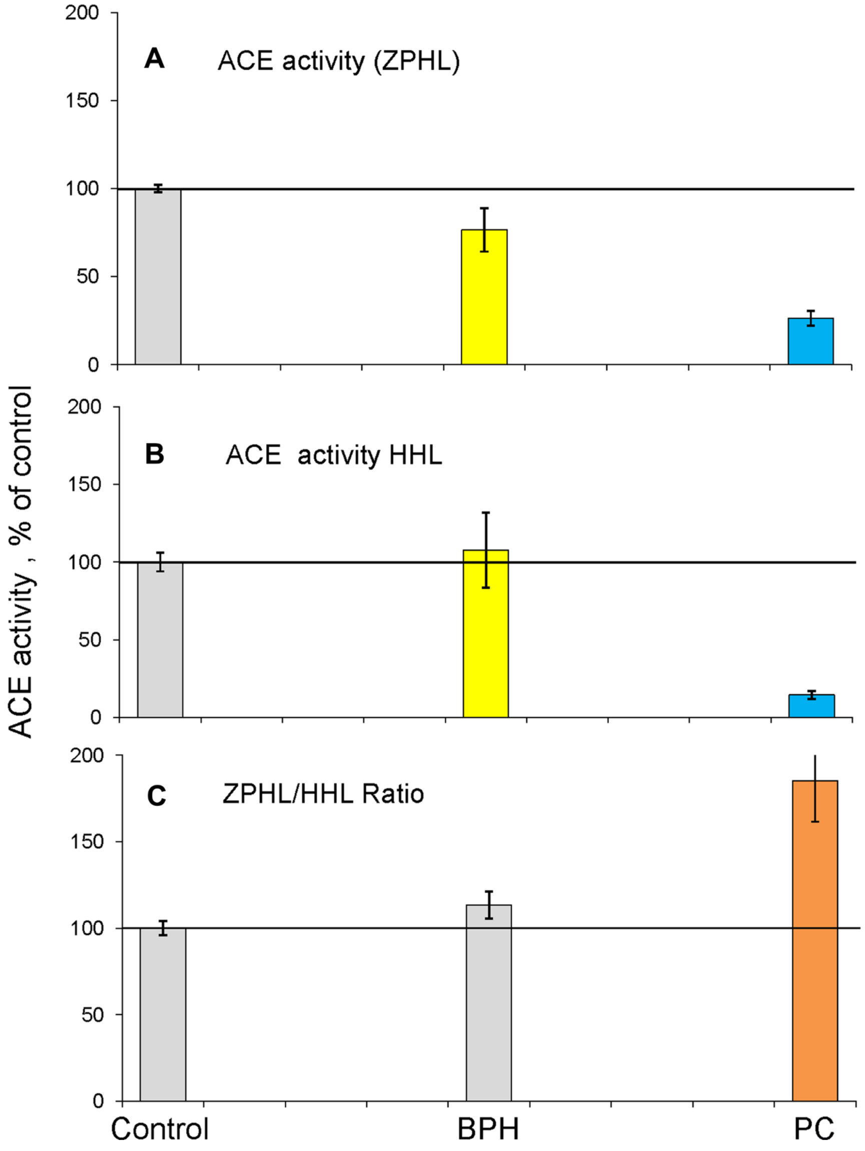

Supplementary Figure 1: Prostate ACE phenotype in different groups of patients (I cohort). Individual values presented in Figure 1 were combined according to their groups. (**A** and **B**) ACE activity with two substrates. (**C**) ZPHL/HHL ratio. Data presented as a mean of at least 2 independent experiments in duplicates. Bars color-coded as in Figure 1.

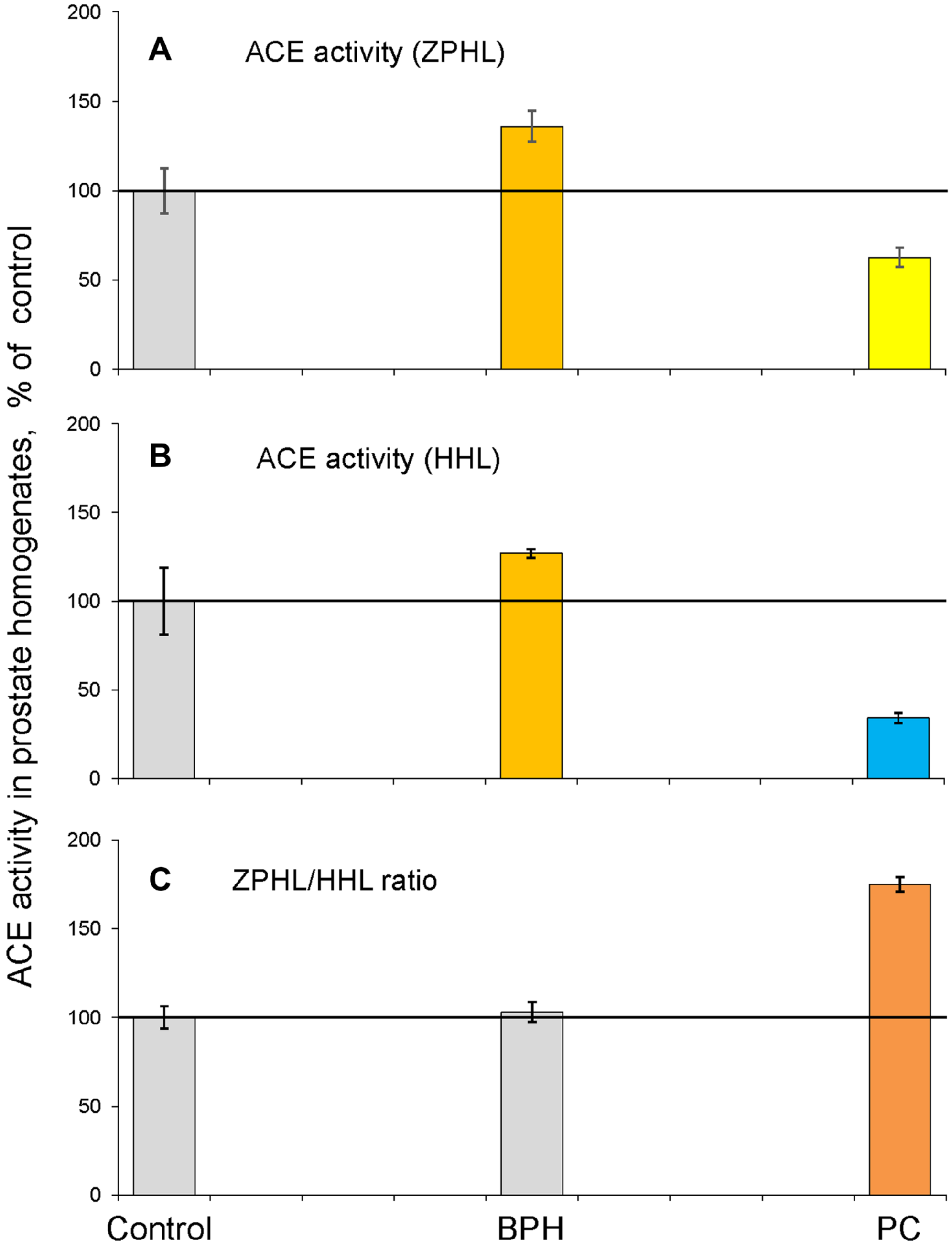

Supplementary Figure 2: Prostate ACE phenotype in different groups of patients (II cohort). Seven needle biopsies from patients with BPH and 7 - from patients with PC (II cohort from Table S1) combined together within each group were used for preparation of prostate tissue homogenates using Potter-Elveheim homogenizer. ACE activity was calculated as mU/mg of protein and expressed as % from mean value of ACE activity in 4 homogenates of unrelated postmortem prostate tissues that were used as a controls (100%). (**A** and **B**) ACE activity with two substrates. (**C**) ZPHL/HHL ratio. Data presented as a mean of at least 2 independent experiments in duplicates. Bars color-coded as in Figure 1.

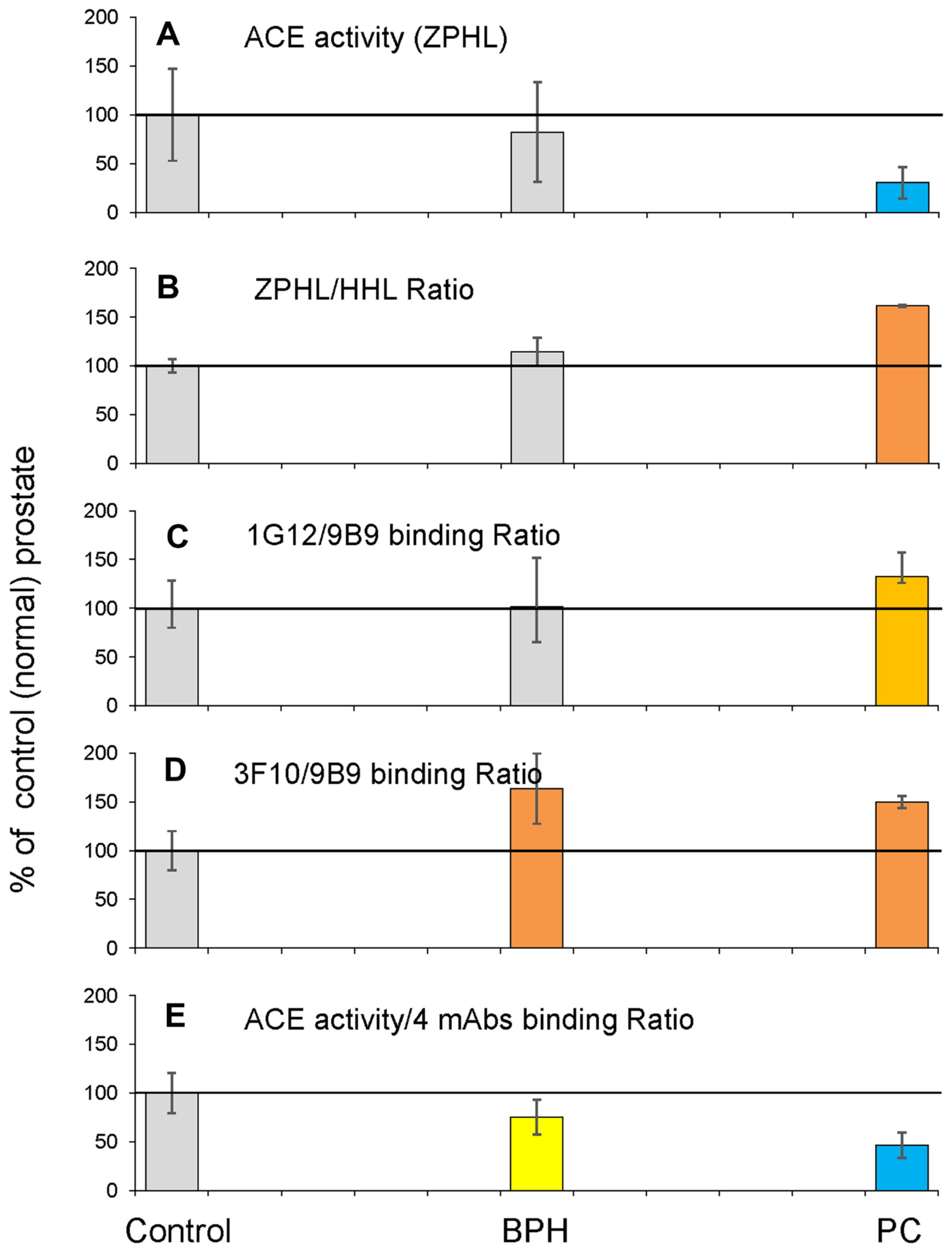

Supplementary Figure 3: Prostate ACE phenotype in different groups of patients (III cohort). Individual values presented in Figure 2 were combined according to their groups. (**A**) ACE activity with ZPHL as a substrate. (**B**) ZPHL/HHL ratio. (**C** and **D**) The ratio of immunoprecipitated ACE activity by mAbs 1G12 or 3F10 to that by mAb 9B9 combined from the values of individual ratios for each group (calculated from data presented on Figure 3). (**E**) ratio of ACE activity to mean of 4 mAbs binding ratios (calculated from data presented on Figure 5) for each group. Data presented as a mean of at least 2independent experiments in duplicates. Bars color-coded- as in Figure 1.

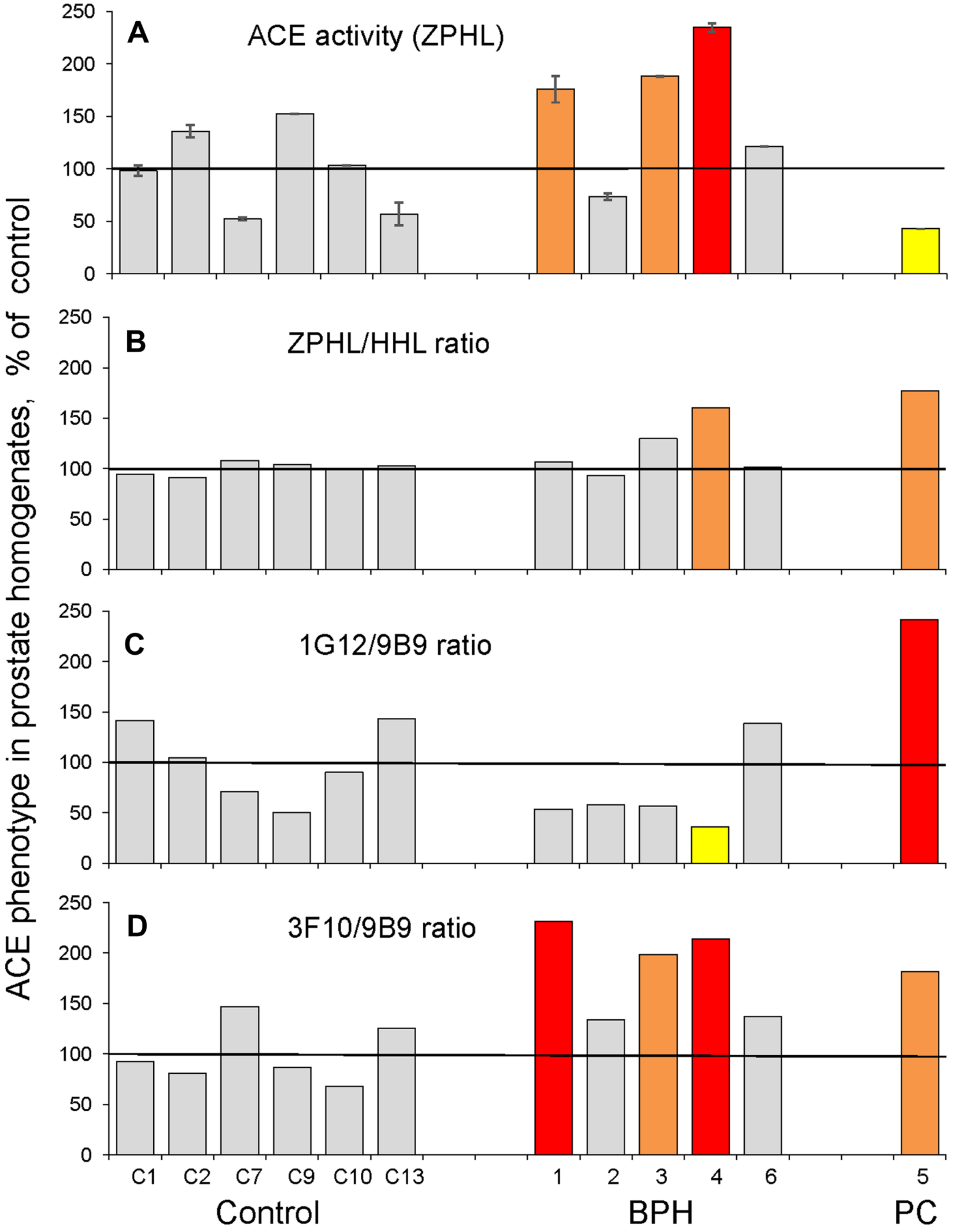

Supplementary Figure 4: Prostate ACE phenotype in different groups of patients (IV cohort). Five needle biopsies from patients with BPH and one from patient with PC were used separately for preparation of prostate tissue homogenates using Speed Mille Plus homogenizer. ACE activity was calculated as mU/mg and expressed as % from mean value of ACE activity in 6 homogenates of unrelated postmortem prostate tissues used as a control (100%). (**A**) ACE activity with ZPHL. (**B**) ZPHL/HHL ratio. (**C** and **D**) The ratio of immunoprecipitated ACE activity by mAbs 1G12 or 3F10 to that by mAb 9B9. Data presented as a mean of at least 2 independent experiments in duplicates. Bars color-coded as in Figure 1.

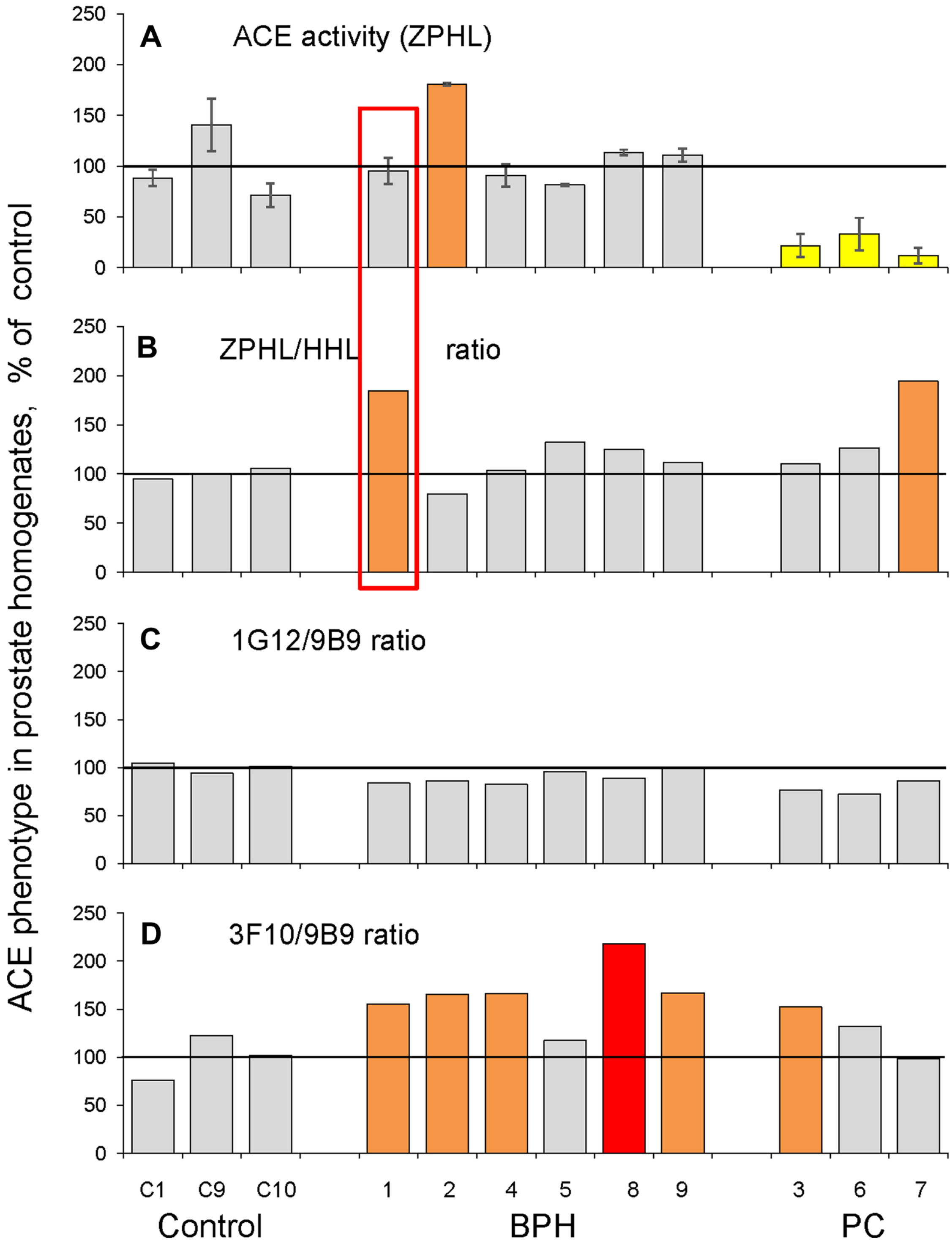

Supplementary Figure 5: Prostate ACE phenotype in different groups of patients (V cohort). Six needle biopsies from patients with BPH and three from patient with PC were used separately for preparation of prostate tissue homogenates using Speed Mille Plus homogenizer. ACE activity was calculated as mU/mg and expressed as % from mean value of ACE activity in 3 homogenates of unrelated postmortem prostate tissues used as a control (100%). (**A**) ACE activity with ZPHL. (**B**) ZPHL/HHL ratio. (**C** and **D**) The ratio of immunoprecipitated ACE activity by mAbs 1G12 or 3F10 to that by mAb 9B9. Data presented as a mean of at least 2 independent experiments in duplicates. Bars color-coded as in Figure 1.
