# Supplementary material for: Tissue ACE phenotyping in prostate cancer
Source: Oncotarget. 2019 Oct 29;10(59):6349–61. doi: 10.18632/oncotarget.27276 (PMC6824872; doi:10.18632/oncotarget.27276)
Supplement: Supplementary file 2 [file oncotarget-10-6349-s002.docx]

**Supplementary Table 1: The designation of patients in different cohorts and description of prostate tissues used for ACE phenotyping**

| Total # | **# within cohort** | **Age,**  years | **PSA,**  ng/m**l** | **Prostate volume, US,** cm^3^ | **Collec- tion method** | **Histology** | **Gleason index** | **PIN grade** | **Remarks.** |
| --- | --- | --- | --- | --- | --- | --- | --- | --- | --- |
| 1 | I-C2 | 37 |  |  | PM | Control |  |  |  |
| 2 | I-C3 | 38 |  |  | PM | Control |  |  |  |
| 3 | I-5 | 71 | 2,0 | 50 | TURP | **BPH** |  |  |  |
| 4 | I-6 | 68 | 2,2 | 42 | TURP | **BPH** |  |  |  |
| 5 | I-7 | 52 | 2,8 | 94 | TURP | **BPH** |  |  |  |
| 6 | I-10 | 76 | 3,5 | 57 | TURP | **BPH** |  | high |  |
| 7 | I-9 | 76 | 5,0 | 50 | TURP | **BPH** |  |  |  |
| 8 | I-8 | 52 | 9,8 | 66 | TURP | **BPH** |  |  |  |
| 9 | I-12 | 75 | 5,7 | 50 | RPE | **PC** | **8** (4+4) |  |  |
| 10 | I-11 | 57 | 8,7 | 50 | RPE | **PC** | **6** (3+3) |  |  |
| 11 | I-13 | 58 | 9,1 | 43 | RPE | **PC** | **7** (3+4) |  |  |
| 12 | I-14 | 67 | N/A | 72 | RPE | **PC** | N/A |  |  |
|  | | | | | | | | | |
| 13 | II-C1 | 32 |  |  | PM | Control |  |  |  |
| 14 | II-C2 | 37 |  |  | PM | Control |  |  |  |
| 15 | II-C3 | 38 |  |  | PM | Control |  |  |  |
| 16 | II-C4 | 33 |  |  | PM | Control |  |  |  |
| 15 | II-7 | 76 | 2,9 | 38 | UGPB | **BPH** |  |  | atrophy |
| 16 | II-13 | 58 | 3,8 | 55 | UGPB | **BPH** |  | low |  |
| 17 | II-12 | 75 | 4,9 | 31 | UGPB | **BPH** |  |  | atrophy |
| 18 | II-1 | 66 | 5,5 | 71 | UGPB | **BPH** |  | low |  |
| 19 | II-10 | 73 | 7,9 | 62 | UGPB | **BPH** |  |  |  |
| 20 | II-11 | 71 | 8,0 | 170 | UGPB | **BPH** |  |  |  |
| 21 | II-2 | 57 | 8,0 | 49 | UGPB | **BPH** |  | high |  |
| 22 | II-8 | 65 | 7,4 | 45 | UGPB | **PC** | **7** (3+4) |  |  |
| 23 | II-6 | 70 | 8,0 | 48 | UGPB | **PC** | **6** (3+3) |  |  |
| 24 | II-14 | 67 | 25,5 | 23 | UGPB | **PC** | **7** (3+4) |  |  |
| 25 | II-9 | 58 | 25,9 | 29 | UGPB | **PC** | **7** (3+4) |  | PNI |
| 26 | II-4 | 75 | 26,0 | 44 | UGPB | **PC** | **7** (4+3) |  |  |
| 27 | II-5 | 69 | 75,0 | 68 | UGPB | **PC** | **8** (4+4) |  | PNI |
| 28 | II-3 | 77 | **135,8** | 89 | UGPB | **PC** | **8** (4+4) |  | PNI |
|  | | | | | | | | | |
| 30 | III-C9 | 39 |  |  | PM | Control |  |  |  |
| 31 | III-10 | 29 |  |  | PM | Control |  |  |  |
| 32 | III-6 | 54 | 1,1 | 52 | UGPB | **BPH** |  |  |  |
| 33 | III-5 | 64 | 4,0 | 189 | UGPB | **BPH** |  |  | atrophy |
| 34 | III-9 | 73 | 7,2 | 73 | UGPB | **BPH** |  |  | prostatitis |
| 35 | III-7 | 69 | 8,5 | 51 | UGPB | **BPH** |  |  | atrophy |
| 36 | III-8 | 58 | 9,0 | 38 | UGPB | **BPH** |  |  | prostatitis |
| 37 | III-3 | 48 | 9,5 | 91 | UGPB | **BPH** |  |  |  |
| 38 | III-4 | 70 | 42,2 | 276 | UGPB | **BPH** |  |  | atrophy |
| 39 | III-1 | 63 | 3,0 | 33 | UGPB | **PC** | **7** (3+4) |  |  |
| 40 | III-2 | 59 | 38,1 | 29 | UGPB | **PC** | **7** (3+4) |  |  |
|  | | | | | | | | | |
| 41 | IV-C1 | 36 |  |  | PM | Control |  |  |  |
| 42 | IV-C2 | N/A |  |  | PM | Control |  |  |  |
| 43 | IV-C7 | 37 |  |  | PM | Control |  |  |  |
| 44 | IV-C9 | 39 |  |  | PM | Control |  |  |  |
| 45 | IV-C10 | 29 |  |  | PM | Control |  |  |  |
| 46 | IV-C13 | 16 |  |  | PM | Control |  |  |  |
| 47 | IV-4 | 60 | 1,8 | 90 | UGPB | **BPH** |  |  |  |
| 48 | IV-2 | 72 | 5,1 | 75 | UGPB | **BPH** |  |  |  |
| 49 | IV-6 | 69 | 5,5 | 80 | UGPB | **BPH** |  |  |  |
| 50 | IV-3 | 82 | 9,0 | 80 | UGPB | **BPH** |  |  |  |
| 51 | IV-1 | 67 | 24,0 | 120 | UGPB | **BPH** |  |  |  |
| 52 | IV-5 | 71 | 8,5 | 70 | UGPB | **PC** | **6** (3+3) |  |  |
|  | | | | | | | | | |
| 53 | V-C1 | 36 |  |  | PM | Control |  |  |  |
| 54 | V-C9 | 39 |  |  | PM | Control |  |  |  |
| 55 | V-C10 | 29 |  |  | PM | Control |  |  |  |
| 56 | V-5 | 61 | 3,8 | 45 | UGPB | **BPH** |  |  |  |
| 57 | V-4 | 59 | 4,8 | 45 | AdE | **BPH** |  |  |  |
| 58 | V-8 | 62 | 5,0 | 70 | UGPB | **BPH** |  |  |  |
| 59 | V-9 | 69 | 5,3 | 50 | UGPB | **BPH** |  |  |  |
| 60 | V-2 | 62 | 7 | 113 | UGPB | **BPH** |  |  |  |
| 61 | V-1 | 82 | 12,7 | 140 | AdE | **BPH** |  |  |  |
| 62 | V-7 | 82 | 7,8 | 107 | UGPB | **PC** | **6** (3+3) |  |  |
| 63 | V-3 | 82 | 9,4 | 60 | UGPB | **PC** | **6** (3+3) |  |  |
| 64 | V-6 | 68 | 17,4 | 50 | RPE | **PC** | **7** (3+4) |  |  |

Abbreviations: PSA - Prostate-Specific Antigen; Grey zone - 2.0-10.0 ng/ml [7];

UGPB - Ultrasound Guided Prostate Biopsy,

RPE - Radical ProstatEctomy, TURP - TransUrethral Resection of the Prostate,

PC - prostate cancer, BPH - benign prostatic hyperplasia, PNI - PeriNeuralInvasion,

PIN - prostatic intraepithelial neoplasia, HPIN - high grade PIN. AdE-adenoectomy,

PM- postmortem
